# Sustained Propranolol Use and Site-Specific All-Cause Mortality after Cancer Diagnosis: A Nationwide Taiwan Cohort Study

**DOI:** 10.32604/or.2026.086291

**Published:** 2026-10-28

**Authors:** Paul J. Chen, Jung-Ju Lin, Wei-Lin Hsu, Der-Cherng Chen, Yu-Hsiang Lin, Chien-Tung Yang, Chao-Hsuan Chen, Yu-Chung Juan, XianXiu Chen

**Affiliations:** 1 Department of Neurosurgery, China Medical University Hospital, Taichung, Taiwan; 2 Neuroscience and Brain Disease Center, China Medical University, Taichung, Taiwan; 3 Sleep Medicine Center, China Medical University Hospital, Taichung, Taiwan

**Keywords:** Propranolol, β-adrenergic blockade, cancer mortality, drug repurposing, pharmacoepidemiology, Taiwan National Health Insurance Research Database, cohort study

## Abstract

**Objectives:** Drug repurposing offers a low-cost route to expanding cancer therapeutics. β-Adrenergic signalling links physiological stress to tumour progression, and propranolol, a non-selective β-blocker, interrupts this pathway upstream. Existing evidence is drawn predominantly from Western, single-cancer studies, and a comprehensive evaluation of survival across the cancer spectrum within a universal-coverage East Asian population has been lacking. **Methods:** In this nationwide retrospective cohort study using Taiwan’s National Health Insurance Research Database, patients with incident cancer (2010–2020) who received propranolol for at least six consecutive months after diagnosis were compared with non-users frequency-matched 1:4 by sex, age, and index year, yielding 11,500 patients (2300 users; 9200 non-users). All-cause mortality was analysed using Cox proportional hazards regression adjusted for age, sex, and area of residence, with a delayed index-date assignment for non-users intended to reduce temporal imbalance between cancer diagnosis and cohort entry. Pre-specified sensitivity analyses comprised alternative exposure-duration thresholds, propensity score matching, and E-value calculation; site-specific analyses applied false discovery rate control. **Results:** Over a median follow-up of 4.9 years, propranolol use was associated with lower all-cause mortality (adjusted hazard ratio [HR] 0.87, 95% confidence interval [CI] 0.80–0.94), corresponding to 6.7 fewer deaths per 1000 person-years. The E-values for the primary adjusted HR and its upper 95% confidence limit were 1.56 and 1.32, respectively. Estimates across alternative exposure-duration thresholds ranged from 0.90 at three months to 0.83 at twelve months. The association was more evident among men and patients aged ≥ 60 years. Among pre-specified grouped categories, lower mortality was associated with propranolol use in genitourinary (HR 0.50, 95% CI 0.32–0.77) and digestive (HR 0.69, 95% CI 0.56–0.85) system cancers, whereas estimates for lung and liver cancer were near unity; individual cancer-site estimates were exploratory and non-significant after correction. **Conclusion:** In this nationwide East Asian cohort, sustained propranolol use was associated with lower all-cause mortality, most evident in genitourinary and digestive system cancers. These associations are hypothesis-generating rather than causal, given the observational design and unmeasured confounders including cancer stage and treatment, and support prioritising these categories for prospective evaluation of propranolol as a candidate for oncological repurposing. Because the exposure definition required survival to completion of a six-month treatment window, residual immortal time and selection bias cannot be excluded and the magnitude of the association may be overstated.

## 1 Introduction

Cancer remains among the leading causes of death worldwide, with an estimated 20.0 million new cancer cases and 9.7 million cancer deaths estimated in 2022 [1]. Despite sustained progress in surgery, systemic therapy, and molecularly targeted treatment, the global cancer burden continues to rise and remains geographically heterogeneous [1]. In this context, drug repurposing—the identification of new oncological applications for medicines with established safety records—offers a resource-efficient route to expanding treatment options, bypassing early-phase development while drawing on well-characterised pharmacology, known adverse-effect profiles, and low cost [2].

Among candidate pathways for repurposing, β-adrenergic signalling has attracted growing attention as a mechanistically coherent link between physiological stress and tumour progression [3,4,5]. Catecholamines released by the sympathetic nervous system activate β-adrenergic receptors on tumour and stromal cells, engaging the cyclic adenosine monophosphate–protein kinase A (cAMP–PKA) cascade and downstream effectors implicated in proliferation, angiogenesis, invasion, and immune evasion [3,4,5]. Propranolol, a non-selective β-blocker in clinical use for more than five decades, competitively blocks these receptors upstream of the entire cascade, providing a single, pharmacologically accessible point of interruption [2,3,4,5]. The translational plausibility of this approach is reflected in regulatory precedent: propranolol received European Union orphan designation for soft tissue sarcoma following reported clinical activity in angiosarcoma [6,7], after its earlier repurposing for infantile haemangioma [8,9].

Epidemiological evidence linking β-blocker use to cancer outcomes has accumulated over the past decade. Barron et al. reported that non-selective β-blocker use was associated with lower breast cancer-specific mortality [10], and subsequent observations were reported in melanoma [11]. A 2018 meta-analysis of 319,006 patients reported substantial heterogeneity across tumour types [12]. More recent systematic reviews and meta-analyses have found no significant overall-survival benefit and have emphasised that the current evidence is predominantly observational and vulnerable to bias and clinical heterogeneity [13,14].

Within Taiwan, two nationwide studies have addressed adjacent questions. Chang et al. reported that long-term propranolol use was associated with a lower risk of incident cancer in a cancer-free population—a contribution to cancer prevention rather than to post-diagnosis survival [15]—whereas a subsequent analysis found no significant association with prognosis in hepatocellular carcinoma [16]. These findings are complementary to, rather than duplicative of, an evaluation of survival across the cancer spectrum, and the divergent hepatocellular result raises the question of whether β-adrenergic blockade is associated with heterogeneous outcomes across tumours with distinct oncogenic drivers [16]. More broadly, cancer incidence patterns and dominant aetiological factors vary geographically [1], supporting direct evaluation in East Asian populations rather than extrapolation from predominantly Western cohorts.

To our knowledge, no study has examined the association between sustained propranolol use and survival across the full spectrum of malignancies within a single, methodologically unified, universal-coverage East Asian cohort of sufficient size and follow-up to characterise tumour-specific heterogeneity. Using Taiwan’s National Health Insurance Research Database, which provides near-complete population coverage, we therefore examined the association between sustained post-diagnosis propranolol use and all-cause mortality in a nationwide cohort of cancer patients. Our objectives were to estimate the overall association in a large, representative East Asian population; to assess whether it is modified by sex and age; and to characterise whether the association differs systematically across cancer site categories, thereby generating evidence to inform the prioritisation and design of future prospective evaluation.

## 2 Materials and Methods

### 2.1 Study Design and Data Source

This nationwide retrospective cohort study used data from Taiwan’s National Health Insurance Research Database (NHIRD), maintained by the National Health Insurance Administration (NHIA) under the Ministry of Health and Welfare [17,18]. Taiwan’s single-payer National Health Insurance (NHI) programme, established in 1995, provides universal coverage to 99% of the country’s 23.74 million residents and contracts with approximately 97% of healthcare providers nationwide [17,19,20]. The NHIRD contains longitudinal, individual-level records of diagnoses, inpatient and outpatient encounters, procedures, and dispensed prescriptions, coded according to the International Classification of Diseases, Ninth Revision, Clinical Modification (ICD-9-CM) [19]. We accessed a validated random sample of 1,000,000 beneficiaries spanning 01 January 2009 to 31 December 2021, a resource extensively used and validated for pharmacoepidemiological research [17,18]. All personal identifiers were encrypted before data release [17]. The study was reported in accordance with the Strengthening the Reporting of Observational Studies in Epidemiology (STROBE) guideline, with the exception of item 13(a): the numbers of individuals screened at the intermediate stages of cohort assembly could not be reported, because under the data-release rules governing the secure analysis environment only aggregate outputs approved for release may be exported, and these intermediate frequencies were not among them. It was approved by the Institutional Review Board of China Medical University Hospital (IRB No. CMUH104-REC2-115), with the requirement for individual informed consent waived given the de-identified nature of the data and the provisions of Taiwan’s Personal Data Protection Act. The study was conducted in accordance with the principles of the Declaration of Helsinki and its subsequent amendments.

### 2.2 Cancer Case Identification

Cancer patients were identified through the Registry of Catastrophic Illness Patient Database (RCIPD), a specialised NHIRD sub-registry. Under NHI regulations, a catastrophic illness certificate for cancer requires pathological confirmation of malignancy and clinical documentation reviewed by at least two independent specialists, conferring high diagnostic validity. We included individuals with an incident cancer diagnosis registered in the RCIPD between 01 January 2010 and 31 December 2020 (ICD-9-CM codes 140–208). Patients with any cancer registration before 01 January 2010 were excluded to restrict the analysis to incident cases and to ensure that propranolol exposure could be characterised unambiguously as post-diagnosis. For the site-specific analyses, malignancies were classified into 20 named individual cancer sites according to the analytic ICD-9-CM coding scheme. Eligible malignant neoplasms within ICD-9-CM codes 140–208 that were not assigned to any of these 20 sites were retained as a predefined residual aggregate category (“other individually unlisted cancer sites”) to preserve complete event accounting.

### 2.3 Exposure Definition and Cohort Assembly

From the cancer population, we identified patients prescribed propranolol (Anatomical Therapeutic Chemical [ATC] code C07AA05) for at least six consecutive months after cancer diagnosis. The date of the first post-diagnosis propranolol prescription defined the index date for exposed patients. The six-month threshold was selected to distinguish sustained use from short courses prescribed for perioperative or situational indications. By definition, patients who did not complete this exposure window, including those who died before completion, were excluded from the exposed cohort; robustness was explored using alternative three- and twelve-month exposure thresholds.

For non-users, the index date was assigned as the cancer diagnosis date plus the median interval between diagnosis and propranolol initiation observed among users. This delayed index-date assignment was intended only to improve temporal alignment of cohort entry; it did not correct the exposure-qualification period. Because exposed status required completion of at least six consecutive months of propranolol treatment while follow-up began at the first post-diagnosis prescription, patients classified as exposed necessarily had to survive and remain treated throughout this post-index exposure-ascertainment period. No equivalent six-month post-index survival or treatment-eligibility requirement was imposed on non-users. The design therefore created asymmetric immortal-time and survivor-selection bias that could favour the propranolol group and exaggerate the apparent reduction in mortality. Accordingly, the findings were interpreted as non-causal associations among selected survivors.

Eligible incident cancer patients were classified into three mutually exclusive groups according to post-diagnosis propranolol exposure: those with at least six consecutive months of propranolol, who formed the exposed cohort; those with no propranolol prescription at any time after diagnosis, who constituted the pool from which the comparison cohort was drawn; and those whose propranolol exposure was of shorter duration, who satisfied neither definition and were excluded from the study. For each exposed patient, four patients from the unexposed pool were randomly selected and frequency-matched on sex, five-year age band, and calendar year of the index date, yielding a 1:4 design. Area of residence was not a matching variable and was instead retained as a covariate in adjusted models. The final analytic cohort comprised 11,500 patients (2300 users and 9200 non-users). All participants were followed from the index date until death, loss to follow-up, or 31 December 2021, whichever occurred first.

### 2.4 Outcome Assessment

The primary outcome was all-cause mortality. Death events were ascertained through deterministic linkage with RCIPD records and hospital admission databases, in which mortality is systematically captured through the mandatory processes governing catastrophic illness certificate termination and discharge-status coding, providing near-complete ascertainment. All-cause rather than cancer-specific mortality was the primary outcome for two reasons: Cause-of-death data coded using the International Classification of Diseases are subject to misclassification, and propranolol’s proposed effects—including modulation of systemic immune function and cardiovascular protection—may influence survival through pathways beyond cancer-specific death.

### 2.5 Covariates

Baseline covariates comprised age at the index date (20–39, 40–49, 50–59, ≥60 years), sex, and area of residence (northern, central, southern, or eastern Taiwan, classified by NHI administrative region and retained as a proxy for socioeconomic status and access to tertiary oncological care). Cancer site category was incorporated as a covariate in propensity score-matched analyses and as a stratification variable in site-specific analyses. For the propensity score-matched analysis, additional covariates were ascertained from the 12 months preceding the index date: pre-specified comorbidities (hypertension, diabetes mellitus, coronary artery disease, heart failure, and chronic liver disease, identified from ICD-9-CM codes in inpatient and outpatient claims) and concurrent medications with putative influence on cancer prognosis (statins, aspirin, and metformin, identified from ATC codes). The NHIRD does not record cancer stage, tumour molecular subtype, or the specific oncological treatments (chemotherapy, radiotherapy, targeted therapy, immunotherapy, or surgery) received during follow-up; these variables were therefore not available for adjustment, a limitation addressed in the Discussion.

### 2.6 Statistical Analysis

Baseline characteristics were compared using the chi-square test for categorical variables. Incidence rates of all-cause mortality were expressed as deaths per 1000 person-years, and absolute rate differences between cohorts were calculated. Cumulative survival was estimated by the Kaplan–Meier method, with 95% confidence intervals derived using the complementary log-log (Greenwood) transformation, and survival distributions were compared using the log-rank test.

The primary measure of association was the hazard ratio (HR) with its 95% confidence interval (CI), estimated by Cox proportional hazards regression. Two models were fitted: Model 1 (unadjusted) and Model 2 (adjusted for age group, sex, and area of residence), with non-users as the reference group. The covariate set of Model 2 was specified to correspond to the frequency-matching design, which matched on sex, five-year age band and index calendar year, with area of residence retained as a covariate because it was not matched. Cancer site, comorbidities and concurrent medications were not matching variables and were not included in Model 2; their joint influence on the overall estimate was instead evaluated in the propensity score-matched sensitivity analysis, in which all three were incorporated into the propensity score model and the matched Cox model. The proportional hazards assumption was assessed using Schoenfeld residuals plotted against follow-up time and was not violated. Stratified analyses by sex and age group were conducted using correspondingly adjusted Cox models.

Cancer site analyses were performed at two levels. Separate Cox models were fitted within each individual cancer site to produce crude and adjusted estimates; given the number of comparisons, the Benjamini–Hochberg procedure was applied to control the false discovery rate at 5%, and these individual-site estimates were treated as exploratory and hypothesis-generating. The aggregate residual category was not included among the comparisons subjected to false discovery rate control, as it does not correspond to a single anatomical site. In addition, two pre-specified grouped categories—genitourinary system cancers and digestive system cancers—were analysed using Cox regression to provide greater precision. Forest plots present adjusted HRs and 95% CIs on a logarithmic scale, with the overall estimate shown as a diamond and subgroup estimates shown as squares. Subgroup markers in the forest plot are of uniform size; statistical precision is represented by the corresponding 95% confidence intervals. For the grouped Kaplan–Meier analysis, the cohort was partitioned into three mutually exclusive categories: genitourinary system cancers, digestive system cancers, and a complementary category comprising all remaining cancer sites not included in the first two categories.

Three pre-specified sensitivity analyses evaluated robustness. First, the minimum exposure threshold was varied to three and twelve consecutive months. Second, a propensity score-matched analysis addressed residual confounding: propensity scores for propranolol use were estimated by multivariable logistic regression incorporating age, sex, area of residence, cancer site category, comorbidities, and concurrent medications, and nearest-neighbour matching was performed using a caliper of 0.2 standard deviations of the logit of the propensity score [21]. Covariate balance was assessed by the absolute standardised mean difference (SMD), with values below 0.10 considered adequate, and the matched association was estimated by a Cox model additionally adjusted for the matched covariates. Third, E-values were calculated for the primary adjusted HR from Model 2 and for the limit of its 95% CI closest to the null, following VanderWeele and Ding [22]. The E-value quantifies the minimum strength of association, on the risk-ratio scale, that an unmeasured confounder would need to have with both propranolol exposure and all-cause mortality, conditional on the measured covariates, to explain away the observed association.

Analyses were performed using Statistical Analysis System (SAS) software, version 9.2 (SAS Institute, Cary, North Carolina, United States). Tests were two-sided, with *p* < 0.05 considered statistically significant before multiple-testing correction; for individual cancer-site analyses, Benjamini–Hochberg-adjusted *p*-values were used.

## 3 Results

### 3.1 Baseline Characteristics and Study Population

Cohort assembly is summarised in Fig. 1. From the random sample of 1,000,000 NHIRD beneficiaries covering 01 January 2009 to 31 December 2021, patients with an incident cancer registered in the RCIPD between 01 January 2010 and 31 December 2020 were identified; those with any cancer registration predating 01 January 2010 were excluded as prevalent cases. Eligible incident cancer patients were then classified according to post-diagnosis propranolol exposure. Patients who received propranolol for fewer than six consecutive months after diagnosis satisfied neither the exposed nor the unexposed definition and were excluded, as were exposed patients who died before completing the six-month exposure-ascertainment window; this survival requirement was applied to the propranolol arm alone. The screening frequencies at these intermediate stages were generated within the secure analysis environment and were not among the aggregate outputs released for export; Fig. 1 therefore presents these steps as explicitly stated eligibility criteria rather than as counts. Every number shown in the diagram is drawn from the analysed dataset. The analytic cohort comprised 11,500 cancer patients—2300 propranolol users and 9200 frequency-matched non-users—in a 1:4 ratio (Fig. 1). Baseline characteristics are shown in Table 1. By design, the cohorts were balanced for age and sex (both *p* = 0.99). The mean age of the population was 61.8 years (standard deviation 13.6), and 55.2% were aged 60 years or older. Women constituted 59.5% of each group. Geographic distribution differed between cohorts (*p* = 0.007), with a higher proportion of users residing in central Taiwan (21.6% versus 18.6%) and a lower proportion in northern Taiwan (43.6% versus 46.2%); area of residence was accordingly retained as a covariate in all adjusted analyses.

**Figure 1:**
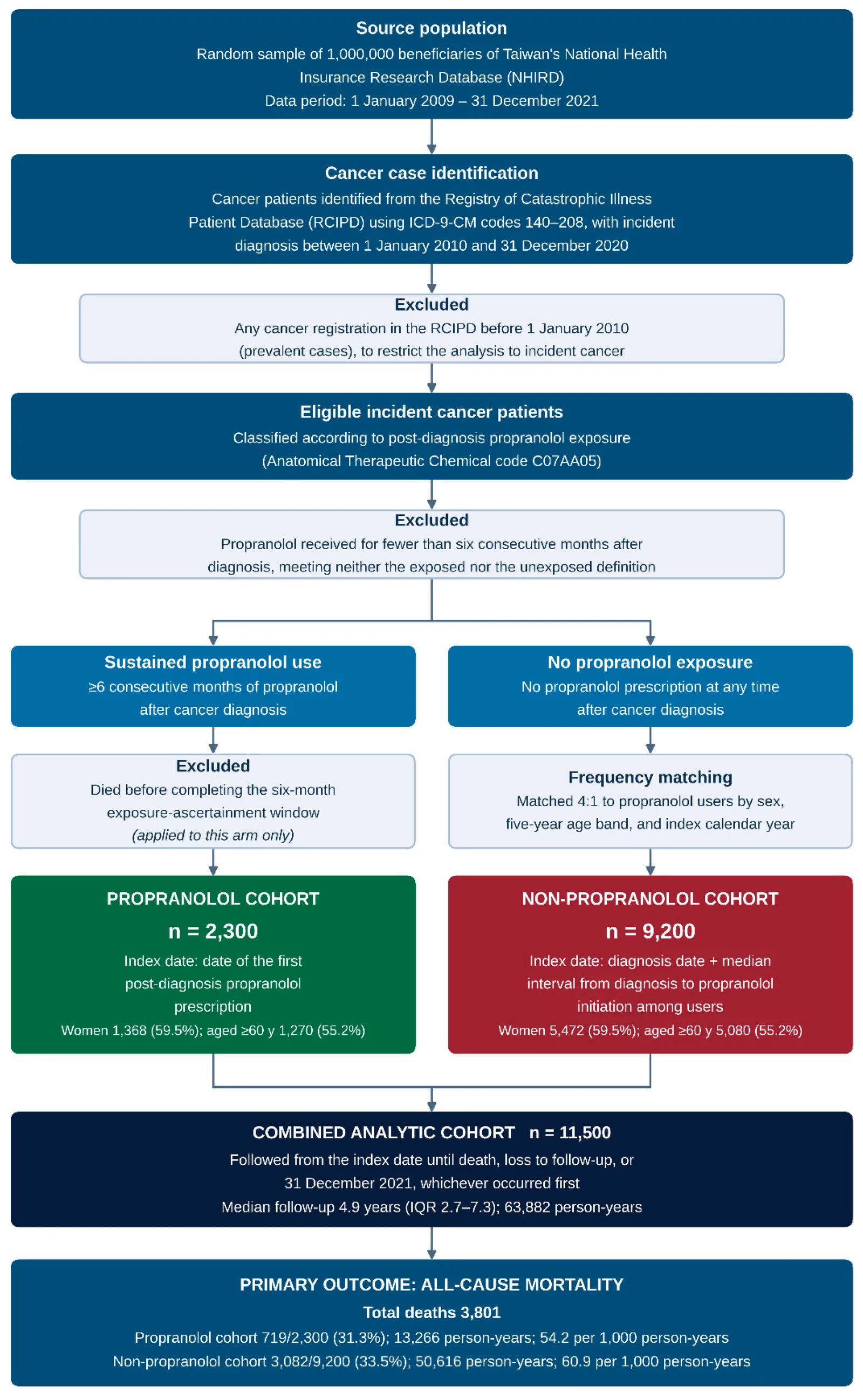
**Patient selection flow diagram.** Stepwise selection of eligible cancer patients from Taiwan’s National Health Insurance Research Database (NHIRD). A random sample of 1,000,000 beneficiaries spanning 01 January 2009 to 31 December 2021 was screened. Cancer patients were identified through the Registry of Catastrophic Illness Patient Database (RCIPD) using ICD-9-CM codes 140–208, with incident diagnoses restricted to 01 January 2010 to 31 December 2020; patients with any cancer registration before 01 January 2010 were excluded to retain incident cases only. Eligible patients were classified into three mutually exclusive groups by post-diagnosis propranolol exposure (ATC code C07AA05); those receiving propranolol for fewer than six consecutive months met neither the exposed nor the unexposed definition and were excluded. The requirement to survive the six-month exposure-ascertainment window was applied to the propranolol cohort only and is shown on that branch accordingly. Non-users were frequency-matched 4:1 to users by sex, five-year age band and index calendar year. Patient numbers are shown at every node for which counts are held in the analytic dataset; the intermediate screening frequencies generated during cohort assembly were not among the aggregate outputs released from the secure analysis environment and are therefore presented as eligibility criteria rather than as counts. Abbreviations and symbols: NHIRD, National Health Insurance Research Database; RCIPD, Registry of Catastrophic Illness Patient Database; ICD-9-CM, International Classification of Diseases, Ninth Revision, Clinical Modification; ATC, Anatomical Therapeutic Chemical classification system; IQR, interquartile range; n, number of patients.

**Table 1:** Baseline demographic characteristics of cancer patients with and without propranolol use.

Variables	Propranolol				*p*-Value
	No		Yes		
	N = 9200		N = 2300		
	n	%	n	%	
**Age, years**					0.99
20–39	688	7.5	172	7.5	
40–49	1384	15.0	346	15.0	
50–59	2048	22.3	512	22.3	
≥60	5080	55.2	1270	55.2	
**Sex**					0.99
Women	5472	59.5	1368	59.5	
Men	3728	40.5	932	40.5	
**Area of residence**					
Northern	4247	46.2	1002	43.6	0.007
Central	1707	18.6	497	21.6	
Southern	2506	27.2	611	26.6	
Eastern	740	8.0	190	8.3	

Note: Data are presented as n (%). N denotes the total number of patients in each exposure group, and n denotes the number of patients in each category. Propranolol users received sustained propranolol treatment (≥6 consecutive months) after cancer diagnosis; non-users were frequency-matched 1:4 by sex, five-year age band, and index calendar year. Between-group differences were assessed using the chi-square test.

### 3.2 Overall Association between Propranolol Use and All-Cause Mortality

Over a median follow-up of 4.9 years (interquartile range 2.7–7.3), representing 63,882 person-years of observation (13,266 among users and 50,616 among non-users), 3801 deaths were recorded: 719 among users and 3082 among non-users (Table 2). The incidence rate of all-cause mortality was lower among users than non-users (54.2 versus 60.9 per 1000 person-years), an absolute difference of 6.7 fewer deaths per 1000 person-years. In the unadjusted analysis (Model 1), propranolol use was associated with lower all-cause mortality (crude hazard ratio [HR] 0.89, 95% confidence interval [CI] 0.82–0.96, *p* < 0.01); after adjustment for age group, sex, and area of residence (Model 2), the association was similar (adjusted HR 0.87, 95% CI 0.80–0.94, *p* < 0.001) (Table 2). Kaplan–Meier curves separated approximately one year after the index date, with users maintaining higher cumulative survival throughout follow-up (log-rank *p* < 0.001) (Fig. 2).

**Figure 2:**
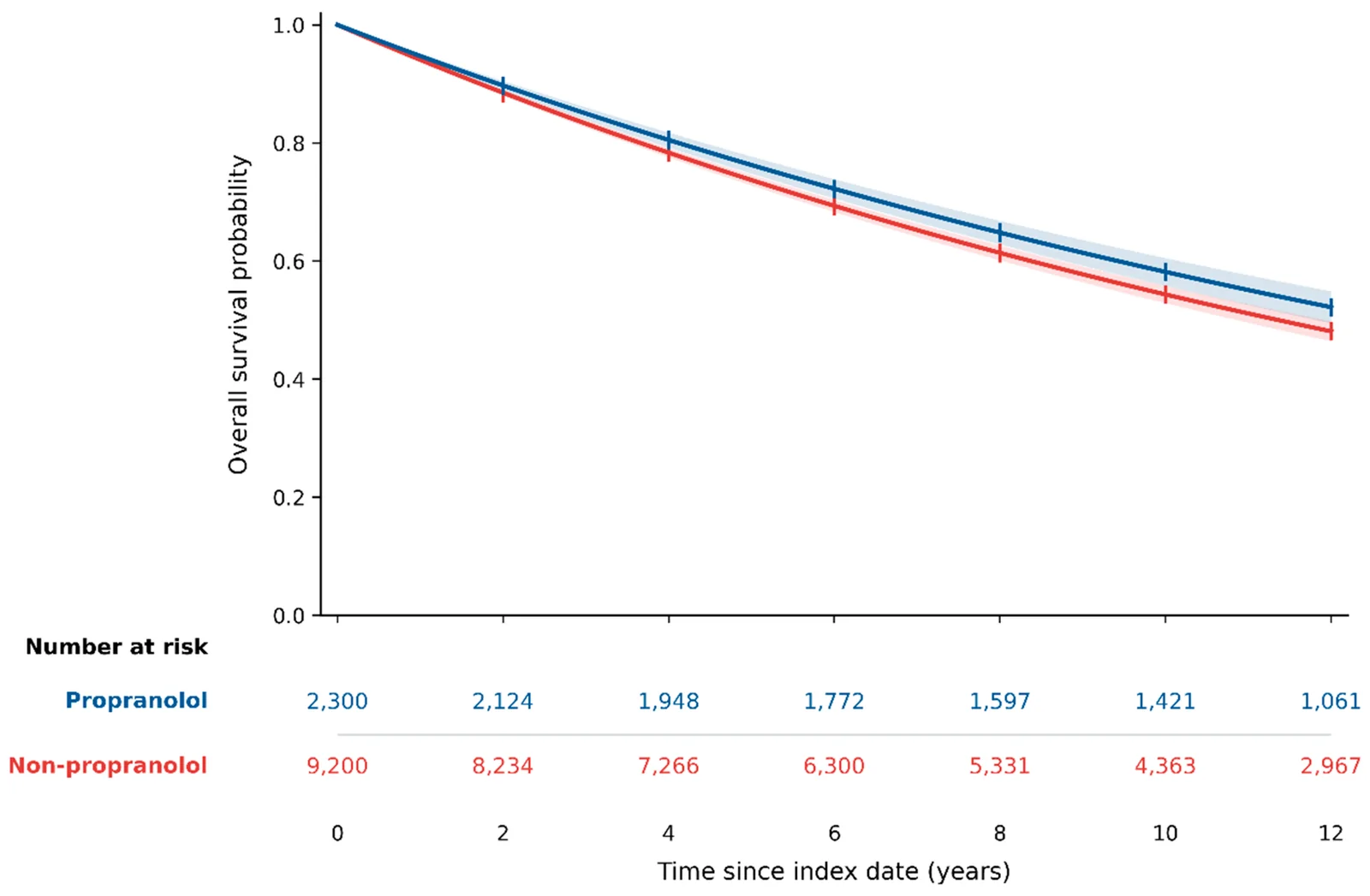
**Kaplan–Meier overall survival curves for propranolol users versus non-users.** Cumulative survival from the index date among propranolol users (n = 2300; blue) and non-users (n = 9200; red). Shaded areas denote 95% confidence intervals; vertical tick marks indicate censoring, and numbers at risk shown at two-year intervals. Survival differed significantly between groups (log-rank *p* < 0.001).

**Table 2:** Absolute mortality, incidence rates and hazard ratios for all-cause mortality among cancer patients with and without propranolol use.

Outcome	Non-users (N = 9200)	Propranolol Users (N = 2300)
Deaths, n/N (%)	3082/9200 (33.5%)	719/2300 (31.3%)
Person-years	50,616	13,266
Incidence rate per 1000 person-years	60.9	54.2
Model 1, crude HR (95% CI)	1.00 (reference)	0.89 (0.82–0.96)**
Model 2, adjusted HR (95% CI)	1.00 (reference)	0.87 (0.80–0.94)***

Note: Model 1 shows unadjusted (crude) hazard ratios; Model 2 shows hazard ratios adjusted for age, sex, and area of residence (Cox proportional hazards regression), with non-users as the reference group (HR = 1.00). The proportional hazards assumption was verified using Schoenfeld residuals. **Abbreviations and symbols:** HR, hazard ratio; CI, confidence interval; N, total number of patients in the exposure group; n, number of deaths. **Significance levels:** **p < 0.01; ***p < 0.001.

### 3.3 Sensitivity Analyses

Results of the three pre-specified sensitivity analyses are summarised in Supplementary Table S1. When the minimum exposure threshold was set to three consecutive months, the association was retained (adjusted HR 0.90, 95% CI 0.83–0.97, *p* < 0.01); at twelve consecutive months, the estimate was 0.83 (95% CI 0.75–0.91, *p* < 0.001). In the propensity score-matched analysis, all covariates achieved adequate balance after matching (absolute standardised mean differences < 0.10; Supplementary Fig. S1A), and the association persisted (adjusted HR 0.85, 95% CI 0.77–0.93, *p* < 0.001; Supplementary Fig. S1B). Because cancer site category, comorbidities and concurrent medications were incorporated into this analysis but not into Model 2, the closeness of the matched estimate to the primary estimate indicates that imbalance in these variables between cohorts did not materially influence the overall association. For the primary Model 2 adjusted HR of 0.87 (95% CI 0.80–0.94), the E-value was 1.56 for the point estimate. The corresponding E-value for the upper 95% confidence limit of 0.94—the limit closest to the null value of 1.00—was 1.32. Thus, an unmeasured confounder would need to be associated with both propranolol use and all-cause mortality by risk ratios of at least 1.56 each, conditional on the measured covariates, to explain away the point estimate; corresponding associations of 1.32 each could move the confidence interval to the null. This analysis addresses unmeasured confounding but does not quantify immortal-time or survivor-selection bias.

### 3.4 Stratified Analyses by Sex and Age

Stratified results are presented in Table 3 and Fig. 3. By sex, the association was statistically significant among men (adjusted HR 0.85, 95% CI 0.76–0.95, *p* < 0.01) and of borderline significance among women (adjusted HR 0.89, 95% CI 0.79–1.00, *p* = 0.05). Among men, the all-cause mortality rates were 77.3 and 88.3 deaths per 1000 person-years among propranolol users and non-users, respectively, corresponding to 10.9 fewer deaths per 1000 person-years among users. Among women, the corresponding rates were 39.8 and 44.5 deaths per 1000 person-years, respectively, corresponding to 4.7 fewer deaths per 1000 person-years among users (Table 3). By age, the association was statistically significant among patients aged 60 years or older (adjusted HR 0.87, 95% CI 0.79–0.96, *p* < 0.01), in whom the incidence rate was 75.4 versus 85.1 per 1000 person-years (absolute difference 9.7). A trend in the same direction was observed at 50–59 years (adjusted HR 0.82, 95% CI 0.67–1.01, *p* = 0.06), while estimates at 40–49 years (adjusted HR 0.97, 95% CI 0.74–1.27) and 20–39 years (adjusted HR 0.88, 95% CI 0.59–1.34) were not statistically significant, with wide confidence intervals corresponding to smaller numbers of events (Table 3).

**Figure 3:**
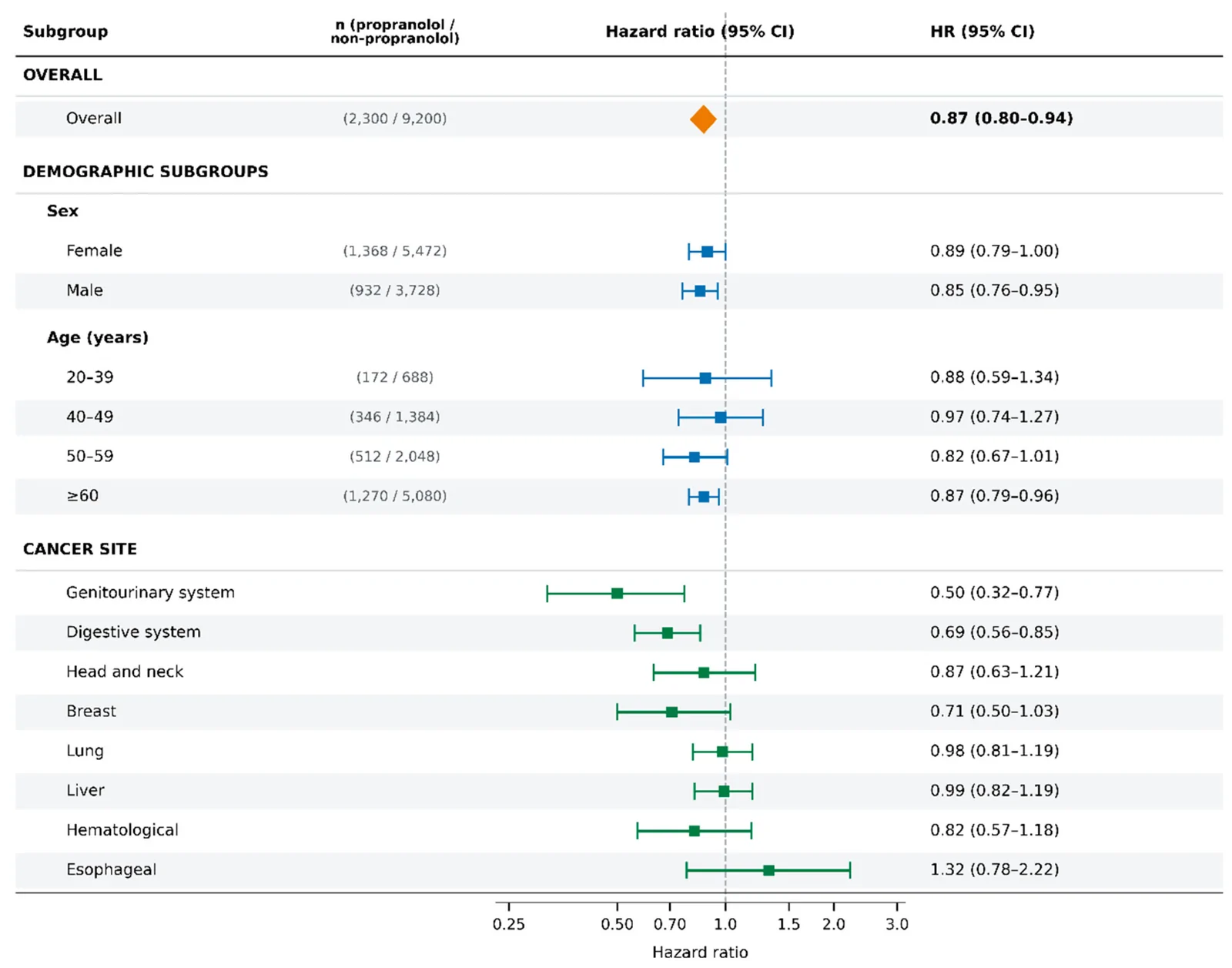
**Forest plot of adjusted hazard ratios for all-cause mortality by demographic subgroup and cancer site category.** Estimates compare propranolol users with non-users. Horizontal lines denote 95% confidence intervals, and the dashed vertical line indicates the null value of 1.0. The diamond represents the overall estimate; blue squares represent demographic subgroups, and green squares represent cancer site categories. Sample sizes are presented as the numbers of propranolol users/non-users. Genitourinary and digestive system cancers were prespecified grouped categories; the selected individual cancer-site estimates are exploratory. Abbreviations and symbols: HR, hazard ratio; CI, confidence interval.

**Table 3:** Absolute mortality, incidence rates, and hazard ratios for all-cause mortality stratified by sex and age group.

**Panel A. Absolute mortality and incidence rates**							
**Subgroup**	**Non-Users Deaths, n/N (%)**	**Non-Users PY**	**Non-Users IR**	**Propranolol Deaths, n/N (%)**		**Propranolol PY**	**Propranolol IR**
Female	1408/5472 (25.7%)	31,649	44.5	326/1368		8184	39.8
				−23.80%			
Male	1674/3728 (44.9%)	18,967	88.3	393/932		5082	77.3
				−42.20%			
20–39 years	125/688 (18.2%)	4596	27.2	28/172		1155	24.2
				−16.30%			
40–49 years	266/1384 (19.2%)	8916	29.8	67/346		2297	29.2
				−19.40%			
50–59 years	524/2048 (25.6%)	11,640	45.0	112/512		3026	37
				−21.90%			
≥60 years	2167/5080 (42.7%)	25,463	85.1	512/1270		6787	75.4
				−40.30%			
**Panel B. Cox proportional hazards regression estimates**							
**Subgroup**	**Model 1, Crude HR (95% CI)**				**Model 2, Adjusted HR (95% CI)**		
Female	0.90 (0.79–1.01)				0.89 (0.79–1.00)		
Male	0.90 (0.78–0.97)				0.85 (0.76–0.95)		
20–39 years	0.90 (0.59–1.34)				0.88 (0.59–1.34)		
40–49 years	1.00 (0.74–1.27)				0.97 (0.74–1.27)		
50–59 years	0.80 (0.67–1.00)				0.82 (0.67–1.01)		
≥60 years	0.90 (0.80–0.97)				0.87 (0.79–0.96)		

**Note:** Panel A presents deaths, person-years, and incidence rates for propranolol users and non-users within each sex and age stratum. Incidence rates were calculated as the number of deaths divided by accumulated person-years and are expressed per 1000 person-years. Panel B presents hazard ratios estimated using Cox proportional hazards regression. Model 1 is unadjusted. In Model 2, sex-specific estimates were adjusted for age group and area of residence, whereas age-specific estimates were adjusted for sex and area of residence. Non-users served as the reference group (HR = 1.00) within each stratum. **Abbreviations and symbols:** CI, confidence interval; HR, hazard ratio; IR, incidence rate per 1000 person-years; N, total number of patients in each subgroup; n, number of deaths; PY, person-years.

### 3.5 Cancer Site-Specific and Grouped Analyses

Estimates for all twenty named individual cancer sites are presented in Table 4, whereas Fig. 3 provides a visual summary of the pre-specified grouped categories and selected individual cancer sites. Across the twenty named individual sites, no estimate reached nominal statistical significance before correction and none retained significance after Benjamini–Hochberg correction; these estimates are reported as exploratory. Table 4 additionally presents an aggregate residual category, “Other individually unlisted cancer sites”, comprising malignancies that do not fall within any named anatomical category (99 deaths among non-users and 24 among users). Patients in this category contributed to the analytic cohort and to all overall and grouped analyses, and their deaths are included in the totals of 3082 and 719; the category was not treated as an individual site and was not counted among the twenty comparisons subjected to false discovery rate control. The largest numerical point estimates below unity at the individual-site level were observed for kidney cancer (adjusted HR 0.59, 95% CI 0.32–1.06), bladder cancer (adjusted HR 0.64, 95% CI 0.36–1.13), and breast cancer (adjusted HR 0.71, 95% CI 0.50–1.03). Lung cancer (adjusted HR 0.98, 95% CI 0.81–1.19) and liver cancer (adjusted HR 0.99, 95% CI 0.82–1.19) showed estimates at or near unity. Prostate cancer (adjusted HR 0.77, 95% CI 0.50–1.18), colorectal cancer (adjusted HR 0.83, 95% CI 0.66–1.04), and stomach cancer (adjusted HR 0.81, 95% CI 0.57–1.15) showed non-significant point estimates below unity, and head and neck cancer (adjusted HR 0.87, 95% CI 0.63–1.21), haematological malignancies (adjusted HR 0.82, 95% CI 0.57–1.18), and pancreatic cancer (adjusted HR 0.89, 95% CI 0.56–1.41) showed non-significant estimates in the same direction. Adjusted point estimates above unity were observed for brain cancer (adjusted HR 1.72, 95% CI 0.70–4.21), oesophageal cancer (adjusted HR 1.32, 95% CI 0.78–2.22), uterine/corpus cancer (adjusted HR 1.24, 95% CI 0.47–3.25), and cervical cancer (adjusted HR 1.06, 95% CI 0.70–1.59). However, all corresponding confidence intervals crossed unity, and none of these estimates reached nominal statistical significance. The residual aggregate category of other individually unlisted cancer sites also had a point estimate above unity (adjusted HR 1.12, 95% CI 0.71–1.76), but it was not considered an individual cancer site or included among the twenty individual-site comparisons subjected to false discovery rate control.

In the two pre-specified grouped analyses (Fig. 3), propranolol use was associated with lower all-cause mortality among patients with genitourinary system cancers (adjusted HR 0.50, 95% CI 0.32–0.77, *p* < 0.01) and among patients with digestive system cancers (adjusted HR 0.69, 95% CI 0.56–0.85, *p* < 0.001). Kaplan–Meier curves for the two pre-specified grouped categories and the complementary remaining cancer-site category are shown in Fig. 4. Separation of the survival curves was most pronounced for genitourinary system cancers (Fig. 4A), followed by digestive system cancers (Fig. 4B). Fig. 4C represents the remaining cancer sites rather than the overall cohort and comprises 960 propranolol users and 3895 non-users, with 300 and 1305 deaths, respectively.

**Figure 4:**
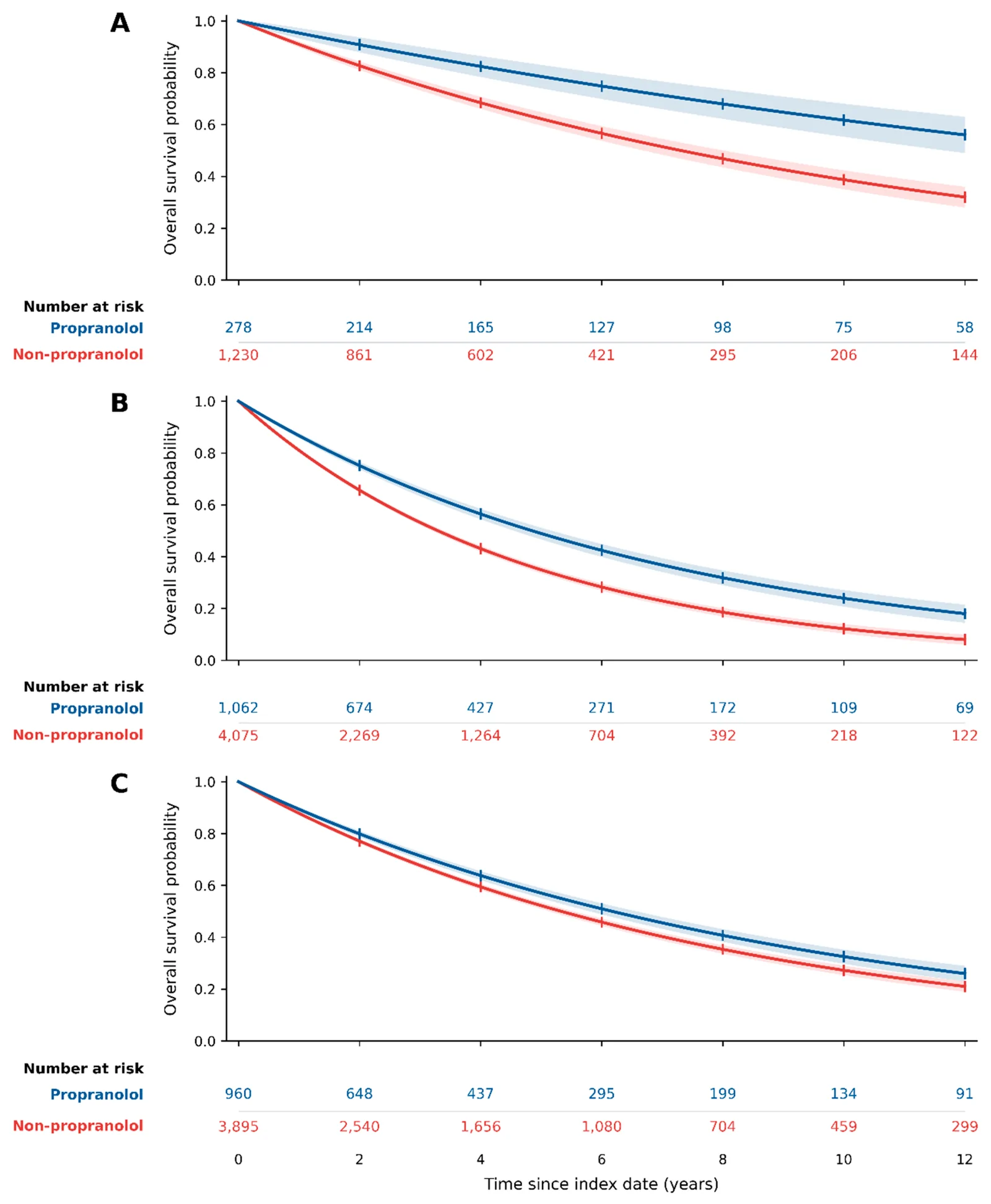
Kaplan–Meier overall survival curves across mutually exclusive cancer-site categories. Cumulative overall survival from the index date among propranolol users and frequency-matched non-users is shown for three mutually exclusive categories: (**A**) genitourinary system cancers, comprising prostate, bladder, kidney, cervical, and uterine/corpus cancers (278 users and 1230 non-users; 87 and 412 deaths, respectively); (**B**) digestive system cancers, comprising colorectal, liver, stomach, pancreatic, oesophageal, and gallbladder/extrahepatic bile duct cancers (1062 users and 4075 non-users; 332 and 1365 deaths, respectively); and (**C**) remaining cancer sites, defined as all sites not included in panels A or B (960 users and 3895 non-users; 300 and 1305 deaths, respectively). Together, these categories include all 2300 propranolol users, 9200 non-users, and 3801 deaths in the analytic cohort. Blue curves represent propranolol users and red curves represent non-users. Shaded areas denote 95% confidence intervals, vertical tick marks indicate censoring, and numbers at risk are shown below each panel at two-year intervals.

**Table 4:** Exploratory analyses of all-cause mortality across 20 individual cancer sites and one residual aggregate category among propranolol users and non-users.

Cancer Site	Propranolol				Model 1	Model 2
	No		Yes			
	Deaths, n	IR	Deaths, n	IR	Crude HR	Adjusted HR
					(95% CI)	(95% CI)
Head and neck cancer	200	3.95	43	3.24	0.88 (0.63, 1.22)	0.87 (0.63, 1.21)
Oesophageal cancer	89	1.76	18	1.36	1.28 (0.77, 2.14)	1.32 (0.78, 2.22)
Stomach cancer	206	4.07	37	2.79	0.82 (0.58, 1.16)	0.81 (0.57, 1.15)
Colorectal cancer	418	8.26	91	6.86	0.82 (0.66, 1.03)	0.83 (0.66, 1.04)
Liver cancer	479	9.46	147	11.08	0.99 (0.82, 1.19)	0.99 (0.82, 1.19)
Gallbladder and extra hepatic bile ducts	62	1.22	14	1.06	0.82 (0.46, 1.46)	0.83 (0.45, 1.52)
Pancreas cancer	111	2.19	25	1.88	0.92 (0.59, 1.42)	0.89 (0.56, 1.41)
Laryngeal cancer	26	0.51	7	0.53	0.89 (0.38, 2.07)	0.81 (0.33, 2.03)
Lung cancer	550	10.87	137	10.33	1.04 (0.86, 1.25)	0.98 (0.81, 1.19)
Melanoma	11	0.22	1	0.08	0.33 (0.04, 2.56)	0.29 (0.03, 2.52)
Skin cancer	30	0.59	4	0.3	0.56 (0.20, 1.59)	0.52 (0.18, 1.49)
Breast cancer	171	3.38	35	2.64	0.77 (0.54, 1.11)	0.71 (0.50, 1.03)
Uterus and corpus cancer	26	0.51	5	0.38	1.15 (0.44, 3.00)	1.24 (0.47, 3.25)
Cervical cancer	135	2.67	28	2.11	1.17 (0.78, 1.76)	1.06 (0.70, 1.59)
Prostate cancer	101	2	27	2.04	0.73 (0.48, 1.12)	0.77 (0.50, 1.18)
Bladder cancer	80	1.58	14	1.06	0.62 (0.35, 1.09)	0.64 (0.36, 1.13)
Kidney cancer	70	1.38	13	0.98	0.57 (0.32, 1.03)	0.59 (0.32, 1.06)
Brain cancer	35	0.69	8	0.6	1.49 (0.69, 3.21)	1.72 (0.70, 4.21)
Thyroid cancer	18	0.36	6	0.45	0.67 (0.26, 1.68)	0.85 (0.33, 2.18)
Hematologic malignancy	165	3.26	35	2.64	0.84 (0.58, 1.21)	0.82 (0.57, 1.18)
Other individually unlisted cancer sites (residual aggregate category)	99	1.96	24	1.81	1.14 (0.73, 1.78)	1.12 (0.71, 1.76)

**Note:** Model 1 shows unadjusted (crude) hazard ratios; Model 2 shows hazard ratios adjusted for age, sex, and area of residence (Cox proportional hazards regression), with non-users as the reference group for every site. The adjustment set for each model is stated here in full, and the column headings therefore carry no footnote symbols. Incidence rates are expressed per 1000 person-years of the whole cohort (50,616 person-years in non-users and 13,266 in users); the site-specific rates shown therefore sum to the overall rates of 60.9 and 54.2 per 1000 person-years reported in Table 2, and are not rates within the site-specific population. “Other individually unlisted cancer sites” is a residual aggregate category comprising all malignancies within ICD-9-CM codes 140–208 that do not fall within any of the twenty named anatomical cancer-site categories; it is not an individual cancer site. Patients in this category were included in the analytic cohort and in all overall, sex-stratified, age-stratified and grouped-category analyses, and their deaths are included in the totals of 3082 and 719. An exploratory model is shown for the category for completeness, but because it is anatomically heterogeneous the estimate is not interpretable as a site-specific effect, and it was not counted among the twenty comparisons subjected to false discovery rate control. The Benjamini–Hochberg procedure was applied across the twenty named individual sites; no estimate reached nominal statistical significance before correction and none retained significance after it, so the inclusion or exclusion of the aggregate category from the correction does not alter any conclusion. Accordingly, and in contrast to Table 2 and Table 3, no significance markers are shown against any estimate in this table; all individual-site estimates are exploratory and hypothesis-generating rather than confirmatory. Abbreviations and symbols: HR, hazard ratio; CI, confidence interval; IR, incidence rate per 1000 person-years; ICD-9-CM, International Classification of Diseases, Ninth Revision, Clinical Modification; n, number of deaths.

## 4 Discussion

### 4.1 Principal Findings

In this nationwide cohort of 11,500 cancer patients followed for a median of 4.9 years, sustained post-diagnosis propranolol use was associated with lower all-cause mortality (adjusted HR 0.87, 95% CI 0.80–0.94). The association persisted after propensity score matching, while the alternative exposure-threshold and E-value analyses provided complementary assessments of robustness. Nevertheless, these analyses do not eliminate the time-related, selection, and unmeasured-confounding concerns inherent in the observational design. The association was more evident among men and patients aged 60 years or older. At the level of pre-specified grouped categories, lower mortality was associated with propranolol use among patients with genitourinary and digestive system cancers, whereas estimates for lung and liver cancer were near unity. Estimates for individual cancer sites did not reach significance after correction for multiple comparisons and are interpreted as exploratory.

### 4.2 Mechanistic Plausibility and β-Adrenergic Receptor Subtypes

The overall association is consistent with the established biological rationale for β-adrenergic blockade in oncology, in which catecholamine-driven activation of protein kinase A and its downstream effectors is attenuated at a pharmacologically upstream point (Supplementary Fig. S2) [3,4,5,23] Three beta-adrenergic receptor subtypes—the beta-1 adrenergic receptor (β1-AR; encoded by *ADRB1*), beta-2 adrenergic receptor (β2-AR; encoded by *ADRB2*), and beta-3 adrenergic receptor (β3-AR; encoded by *ADRB3*)—differ in tissue distribution and expression across tumour types. β2-AR is the most consistently expressed subtype in epithelial malignancies and the principal mediator implicated in catecholamine-driven tumour signalling, whereas β1-AR and β3-AR are expressed more variably [3,4]. Propranolol is a non-selective antagonist of β1-AR and β2-AR, with minimal activity at β3-AR; consequently, the degree to which a given tumour depends on β1/β2-mediated signalling, rather than on β-independent oncogenic drivers, provides a plausible framework for the heterogeneity observed across cancer categories.

Within this framework, prostate tumours exhibit functionally relevant crosstalk between adrenergic and androgen-receptor signalling [24,25]. Recent experimental work further showed that propranolol induced pro-apoptotic signalling and inhibited mitogen-activated protein kinase activity in prostate and bladder cancer cell models [26]. The digestive group is similarly contextualised by dense enteric innervation and recent colorectal models showing that propranolol can modulate antitumour immunity and treatment response, although these findings remain preclinical or biomarker-based [27,28,29,30]. The near-unity estimates for lung and liver cancer may reflect the importance of oncogenic programmes such as epidermal growth factor receptor (EGFR)-mutant and anaplastic lymphoma kinase (ALK)-rearranged lung adenocarcinoma [31,32] and hepatitis B virus-driven hepatocarcinogenesis [33]. Conversely, recent experimental studies indicate that β-adrenergic signalling can influence HepG2 proliferation, macrophage polarisation in hepatoma, and murine lung tumour progression [34,35,36]. The discrepancy between these experimental observations and the present near-null clinical estimates underscores that preclinical plausibility does not establish a survival effect. The liver cancer result is also consistent with the prior Taiwanese analysis [16]. Differences in sympathoadrenal reactivity by sex and age may provide additional context [37,38,39].

#### 4.2.1 Comparison with Existing Literature

These findings extend the existing pharmacoepidemiological evidence in several respects. Barron et al. reported an association between non-selective β-blocker use and lower breast cancer-specific mortality in a Western population [10], whereas the 2018 meta-analysis by Na et al. reported substantial cross-tumour heterogeneity [12]. More recent syntheses found no significant overall-survival benefit and emphasised the predominantly observational nature of the available evidence [13,14]. The breast cancer estimate observed here (adjusted HR 0.71, 95% CI 0.50–1.03), although not statistically significant, is directionally compatible with earlier reports [10]. However, a 2022 population-based cohort study and meta-analysis found no overall association with breast cancer-specific survival, with a possible association confined to triple-negative disease [40]. Where prior studies largely examined individual tumour types across heterogeneous designs, the present analysis evaluates multiple cancer categories within one internally consistent framework in a universal-coverage East Asian population. The present study is also complementary to the two previous Taiwanese studies, which addressed cancer incidence [15] and hepatocellular carcinoma prognosis [16], respectively.

#### 4.2.2 Pharmacogenomic and Pharmacokinetic Considerations in East Asian Populations

Propranolol is metabolised partly by cytochrome P450 2D6 (CYP2D6) and cytochrome P450 1A2 (CYP1A2), and reduced-function CYP2D6 alleles, including CYP2D6*10, are more frequent in East Asian populations [41,42]. However, the 2024 Clinical Pharmacogenetics Implementation Consortium guideline found actionable CYP2D6 evidence primarily for metoprolol and insufficient evidence to support prescribing recommendations based on ADRB2 genotype or CYP2D6 genotype for other β-blockers [41]. Functional ADRB2 haplotypes can alter receptor expression and responsiveness [43], but whether these differences modify the association between propranolol and cancer survival remains unknown. These pharmacogenomic differences therefore provide a rationale for population-specific evaluation, rather than evidence of an established propranolol-specific pharmacogenomic effect.

#### 4.2.3 Duration-Response and Interpretation

A monotonic pattern was observed across exposure thresholds, with estimates of 0.90, 0.87, and 0.83 at three, six, and twelve months, respectively. However, qualification at successively longer exposure thresholds also required progressively longer survival and treatment persistence. Consequently, this pattern cannot be distinguished from survivor selection, residual immortal-time bias, and a healthy-adherer effect, and should not be interpreted as evidence of a pharmacological duration–response relationship or as strengthening causal inference. The duration-stratified estimates are therefore exploratory and require confirmation using time-varying exposure methods or a properly aligned landmark design, such as within a target trial emulation framework [44].

### 4.3 Clinical and Translational Implications

Propranolol is among the most widely used cardiovascular medicines, with a safety profile accumulated over more than five decades, a generic cost, and near-universal availability. The translational interest in repurposing it for oncology is reflected in regulatory precedent: the European Medicines Agency granted orphan designation for soft tissue sarcoma in 2016 on the basis of activity in angiosarcoma [6,7], a highly vascular tumour, following propranolol’s earlier repurposing for infantile haemangioma [8,9]. For patients already receiving propranolol for established indications at the time of cancer diagnosis, the present findings provide real-world evidence of an association between continued use and lower all-cause mortality. They do not, however, support initiating propranolol for cancer indications outside a clinical trial.

The grouped associations observed here are most useful for prioritising prospective evaluation. Genitourinary and digestive system cancers represent reasonable candidate categories for prospective evaluation, with prostate and bladder cancer of mechanistic interest based on the available adrenergic evidence [24,25,26], and colorectal cancer supported by emerging experimental and biomarker studies [27,28,29,30], time-varying exposure, or target-trial emulation methods that align eligibility, treatment assignment, and the start of follow-up between comparison groups [44]. From a global health perspective, an inexpensive and widely available agent that could be evaluated as an adjunct across multiple tumour types would be of particular relevance to settings with limited access to precision oncology—provided that the present associations are confirmed in prospective, adequately controlled studies.

## 5 Limitations

This study has several important limitations. First, as in all observational pharmacoepidemiology, residual confounding cannot be excluded. The principal concern is indication bias: patients receiving propranolol for cardiovascular or anxiety-related indications may differ from non-users in cardiovascular disease burden, hypertension severity, psychological morbidity, and the intensity of medical surveillance, any of which could independently influence survival. The propensity score-matched analysis (adjusted HR 0.85) partially addresses this through adjustment for comorbidities and concurrent cardiovascular medications, and the E-values—1.56 for the primary Model 2 adjusted HR of 0.87 and 1.32 for its upper 95% confidence limit of 0.94, the limit closest to the null—indicate that unmeasured confounding of relatively modest magnitude could move the point estimate or its confidence interval towards the null. These calculations address unmeasured confounding only and do not account for immortal-time bias, survivor-selection bias, or other time-related design limitations; residual confounding from unmeasured cardiovascular treatment intensity, psychological factors, and lifestyle therefore remains possible. A further indication warrants specific mention: in Taiwan, non-selective β-blockade is widely prescribed for prophylaxis of variceal bleeding in cirrhotic portal hypertension, so propranolol users within the digestive-cancer group are likely to be enriched for cirrhosis, an adverse prognostic marker. This would be expected to bias the digestive and hepatic estimates toward or above the null rather than below it, and may contribute to the near-unity estimate observed for liver cancer.

Second, the database does not record cancer stage, tumour grade, or molecular subtype. Because stage at diagnosis strongly influences both prognosis and prescribing patterns, its absence is a substantive potential confounder that the available methods cannot fully address. Third, and relatedly, the specific oncological treatments received during follow-up—chemotherapy, radiotherapy, targeted therapy, immunotherapy, and surgery—were not available and could not be included in any model; differences in treatment between users and non-users could therefore contribute to the observed association. Fourth, lifestyle factors including smoking, alcohol use, body-mass index, physical activity, and diet, as well as cancer recurrence, were not captured and could not be adjusted for. Fifth, all-cause rather than cancer-specific mortality was the outcome. Mortality was ascertained from the termination of catastrophic illness certificates and from hospital discharge status, which establish the fact and date of death with near-complete coverage but do not record the underlying cause; validated cause-of-death information was not available within the approved data-use protocol, and deaths could therefore not be partitioned by cause. A competing-risk framework, in which death from non-cancer causes is treated as a competing event for cancer-specific death rather than as a censoring event [45], would allow the cause-specific component of the association to be isolated, and represents an informative extension of this work in a dataset with linked death-certificate data. The use of all-cause mortality has the advantage of avoiding the misclassification inherent in administrative cause-of-death coding, but it may dilute tumour-specific associations, biasing site-specific estimates toward the null and suggesting that any true cancer-specific association may be underestimated. Sixth, medication adherence could not be verified, as dispensing records capture prescriptions issued rather than medication consumed; non-differential misclassification of this kind would also be expected to attenuate the association. Seventh, diagnoses were coded throughout in ICD-9-CM, and although this was applied consistently across the observation window, coding imprecision after Taiwan’s 2016 transition cannot be entirely excluded. Eighth, several individual cancer sites, particularly rarer malignancies and the youngest age strata, were characterised by small numbers of events, wide confidence intervals, and limited power. The absence of significance for any individual site after Benjamini–Hochberg correction reflects these constraints, and the above-unity point estimates for brain, oesophageal, uterine/corpus, and cervical cancers should be regarded as exploratory rather than as evidence of increased mortality. Ninth, cancer site was not a matching variable and was not included in the primary model, which was specified to correspond to the matched design. The distribution of cancer sites differed modestly between cohorts, with propranolol users somewhat more likely to have digestive-system cancers and less likely to have genitourinary cancers; because digestive-system cancers carry the higher mortality of the two, this imbalance would be expected to bias the primary estimate toward the null rather than away from it. Consistent with this, the propensity score-matched analysis, which included cancer site category among its covariates, produced a slightly stronger association (0.85) than the primary model (0.87). Third, the database does not record cancer stage, tumour grade, or molecular subtype. Finally, classification as exposed required completion of six consecutive months of propranolol treatment, whereas follow-up began at the first post-diagnosis prescription. Exposed patients therefore had to survive and remain treated throughout the exposure-ascertainment period, and patients who died before its completion could not enter the exposed cohort. Although the delayed index-date assignment for non-users was intended to reduce temporal imbalance before cohort entry, it did not impose an equivalent post-index survival requirement and therefore did not eliminate immortal-time or survivor-selection bias. These biases may have exaggerated the apparent mortality reduction, and an artifactual component of the observed association cannot be excluded. Accordingly, the estimates should be interpreted as non-causal associations observed among selected survivors and require confirmation in studies specifically designed to address time-related bias.

## 6 Conclusion

In this nationwide Taiwanese cohort, sustained post-diagnosis propranolol use was associated with lower all-cause mortality, with the strongest grouped associations observed in genitourinary and digestive system cancers. However, the observational design, the absence of information on cancer stage and treatment, and the asymmetric requirement that exposed patients survive the six-month exposure-ascertainment period preclude causal interpretation and may have exaggerated the apparent mortality reduction. Individual cancer-site estimates were imprecise, and none remained statistically significant after correction for multiple comparisons. These findings should therefore be considered hypothesis-generating and require confirmation in prospective studies or observational analyses using aligned landmark or time-varying exposure designs before propranolol is recommended for oncological use.

## Data Availability

The data supporting the findings of this study were obtained from the Taiwan National Health Insurance Research Database, maintained by the National Health Insurance Administration, Ministry of Health and Welfare. Owing to legal restrictions on the protection of electronic personal data, the datasets are not publicly available, but qualified researchers may apply for access through the National Health Insurance Administration, Ministry of Health and Welfare, Taiwan. The analytical code and statistical scripts are available from the corresponding author upon reasonable request and subject to institutional approval. Analyses were conducted within a secure analysis environment from which only aggregate outputs approved for release may be exported. The intermediate screening frequencies generated during cohort assembly were not among the released outputs and cannot be reproduced without a further data-access application.

## Supplementary Materials

The supplementary material is available online at https://www.techscience.com/doi/10.32604/or.2026.086291/s1.

## Abbreviations

Abbreviation or symbol	Definition
ADRB1/ADRB2/ADRB3	beta-1/beta-2/beta-3 adrenergic receptor genes
ALK	anaplastic lymphoma kinase
AR	adrenergic receptor
ATC	Anatomical Therapeutic Chemical classification system
β1-AR/β2-AR/β3-AR	beta-1/beta-2/beta-3 adrenergic receptors
cAMP	cyclic adenosine monophosphate
CI	confidence interval
CREB	cAMP response element-binding protein
CYP1A2/CYP2D6	cytochrome P450 1A2/cytochrome P450 2D6
EGFR	epidermal growth factor receptor
ERK	extracellular signal-regulated kinase
FAK	focal adhesion kinase
Gs	stimulatory G protein
GU	genitourinary
HR	hazard ratio
ICD-9-CM	International Classification of Diseases, Ninth Revision, Clinical Modification
ICMJE	International Committee of Medical Journal Editors
IQR	interquartile range
IR	incidence rate
IRB	Institutional Review Board
MAPK	mitogen-activated protein kinase
N	total number of patients in the relevant cohort or subgroup
n	number of patients or events, as specified in the corresponding figure or table
NHI	National Health Insurance
NHIA	National Health Insurance Administration
NHIRD	National Health Insurance Research Database
NS	not significant
p	probability value
PKA	protein kinase A
PK/PD	pharmacokinetics/pharmacodynamics
PSM	propensity score matching
PY	person-years
RCIPD	Registry of Catastrophic Illness Patient Database
SAS	Statistical Analysis System
SD	standard deviation
sHR	subdistribution hazard ratio
SMD	standardised mean difference
STROBE	Strengthening the Reporting of Observational Studies in Epidemiology
