# Supplementary material for: Sustained Propranolol Use and Site-Specific All-Cause Mortality after Cancer Diagnosis: A Nationwide Taiwan Cohort Study
Source: Oncol Res. 2026 Oct 28;34(11):15. doi: 10.32604/or.2026.086291 (PMC13732343; doi:10.32604/or.2026.086291)
Supplement: Supplementary file 1 [file OncolRes-34-86291-s001.zip › TSP_OR_86291-Table-S1.docx]

**Supplementary Table S1. Sensitivity analyses evaluating the association between sustained propranolol use and all-cause mortality among cancer patients.**

| **Analytic approach** | **Statistic** | **Estimate** | **95% CI** | **p value** |
| --- | --- | --- | --- | --- |
| **A. Alternative exposure-duration thresholds** |  |  |  |  |
| Primary analysis: propranolol use >=6 consecutive months after cancer diagnosis | Adjusted HR | 0.87 | 0.80-0.94 | <0.001 |
| Alternative threshold: propranolol use >=3 consecutive months after cancer diagnosis | Adjusted HR | 0.90 | 0.83-0.97 | <0.01 |
| Alternative threshold: propranolol use >=12 consecutive months after cancer diagnosis | Adjusted HR | 0.83 | 0.75-0.91 | <0.001 |
| **B. Propensity score-matched analysis** |  |  |  |  |
| Nearest-neighbour PSM with caliper = 0.2 SD of the logit propensity score; Cox model additionally adjusted for cancer type, comorbidities, and concurrent medications | Adjusted HR | 0.85 | 0.77-0.93 | <0.001 |
| **C. Quantification of unmeasured confounding** |  |  |  |  |
| E-value for the primary adjusted point estimate | E-value | 1.56 | - | - |
| E-value for the confidence-limit closest to the null | E-value | 1.32 | - | - |

Footnote: Sensitivity analyses were conducted to assess whether the observed association between sustained propranolol use and all-cause mortality was consistent across alternative exposure definitions and analytic approaches. Cox proportional hazards models were adjusted for age group, sex, and area of residence unless otherwise specified. The propensity score-matched analysis additionally accounted for cancer type, comorbidities, and concurrent medications. E-values were calculated for the primary adjusted hazard ratio and for the confidence-limit closest to the null. For a protective hazard ratio below 1.00, the E-value was calculated using the inverse of the hazard ratio. The E-value for the primary adjusted estimate indicates that an unmeasured confounder would need to be associated with both sustained propranolol use and all-cause mortality by a risk ratio of at least 1.56-fold each, beyond measured covariates, to fully explain the observed point estimate. The corresponding E-value for the confidence-limit closest to the null was 1.32. Abbreviations: Adj. HR, adjusted hazard ratio; CI, confidence interval; SD, standard deviation; PSM, propensity score matching
